# The Determination of Estradiol to Cumulus Oocyte Complex (COC) Number Ratio: Does it Predict the Outcomes of ART Cycles?

**Published:** 2020

**Authors:** Fatemeh Taheri, Marjan Omidi, Mohammad Ali Khalili, Azam Agha-Rahimi, Mojdeh Sabour, Azita Faramarzi, Esmat Mangoli

**Affiliations:** 1- Research and Clinical Center for Infertility, Yazd Reproductive Sciences Institute, Shahid Sadoughi University of Medical Sciences, Yazd, Iran; 2- Fertility and Infertility Research Center, Health Technology Institute, Kermanshah University of Medical Science, Kermanshah, Iran

**Keywords:** E2/oocyte ratio, Estradiol level, IVF, Live birth rate

## Abstract

**Background:**

The aim of this study was to assess the impact of total serum E2 on the day of human chronic gonadotropin (hCG) administration and the serum E2 per oocyte ratio on the outcomes of assisted reproductive technology (ART) cycles.

**Methods:**

A total of 205 women were categorized into 3 groups according to the serum E2 levels: 1: ≤1500 *pg/ml*; 2: 1500–3000 *pg/ml*; 3: >3000 *pg/ml*. Another categorization included 3 groups according to E2/oocyte ratio: A: ≤150 *pg/ml* per oocyte; B: 150–200 *pg/ml* per oocyte; and C: >200 *pg/ml* per oocyte. The outcome compared between groups included laboratory and clinical characteristics. One-way analysis of variance (ANOVA), chi-square and Kruskal-Wallis, and multiple logistic regression model were performed, and appropriate differences were considered significant at p<0.05.

**Results:**

There was a significant difference between the groups based on the E2 levels with respect to laboratory parameters. In group C, the rates of chemical pregnancy (54.1%), clinical pregnancy (50%) and live birth (45.8%) were significantly higher, when compared to other groups. Moreover, according to E2/oocyte ratio, the rate of live birth was higher in group C compared with group A (18.3%, p=0.04), and group C (29.7%, p<0.0001). Logistic regression showed the number of good quality embryos was a positive predictor for live birth (odds ratio=2.03, 95% CI=1–4.1), but the level of E2 on day of HCG was a negative predictor (odds ratio=0.99, 95% CI=0.99–1).

**Conclusion:**

Supraphysiological levels of E2 had no adverse effects on the quality of the embryos in IVF cycles, but may have adverse effect on live birth in fresh transfer. Also, it is confirmed that both the pregnancy and live birth rates were elevated with E2/oocyte ratio ≥200 *pg/ml*.

## Introduction

Controlled ovarian hyperstimulation (COH) is a key factor for employment of a large group of oocytes, in order to increase the chance of pregnancy. The serum estradiol (E2) level increases in the ovarian stimulation program (1). It also plays an important role in the oocytes and follicular maturation, and preparation of the uterus for the implantation (2). The physiological impact of an elevated E2 on the quality of the cycle remains debatable (3–5). The high level of E2 on the day of hCG administration might cause better IVF-ICSI outcomes, or an unfavorable outcome which is caused by disrupted endometrial receptivity (6–8).

Some studies showed that elevated E2 levels on the hCG day correlate with lower pregnancy and implantation rates in IVF cycles (9, 10). However, others claim no such effects (11, 12). On the other hand, usually serum E2 levels on the day of hCG administration is a predictor of the approximate number of cumulus oocyte complex (COC); but, the optimal E2/oocyte ratio has not been well defined (13). Loumaye et al. (14) declared that E2/oocyte ratio is the strong parameter for predicting success in IVF programs. It was shown that the highest rate of pregnancy was obtained at an E2/oocyte ratio of 70–140 *pg/ml*/oocyte. Other studies reported different results of pregnancy and implantation in the presence of different E2/oocyte ratios. In addition, the low rate of pregnancy was obtained in the presence of elevated E2/oocyte ratios (15). However, Orvieto et al. (16) did not observe a difference in the pregnancy rates at different E2/oocyte ratios in GnRH-agonist protocols. Due to these inconclusive results regarding the effects of E2 levels on the day of hCG administration, and undetermined E2/oocyte ratio on ART outcomes, this study was designed to investigate the relationship between different levels of E2 and different E2/oocyte ratios with the embryo quality and the clinical outcomes in ART cycles using GnRH antagonist.

## Methods

### Patients:

The permission to perform this study was given by the ethics committee of Institute for Reproductive Sciences, Yazd, Iran. In this retrospective study, the clinical records of 205 women who referred to our institute were reviewed. Patients with a normal ovarian reserve who received GnRH antagonist protocol were included in this study. The patients with poor ovarian reserve, or apparent endometrial pathology, advanced maternal age, severe male factor infertility, and other stimulation protocols were excluded. Patients were divided into three groups according to the E2 levels on the day of hCG administration: Group A: ≤1500 *pg/ml*; Group B: 1500–3000 *pg/ml*; Group C: >3000 *pg/ml*. They were also divided into groups according to their peak of E2/oocyte ratio: Group A: ≤150 *pg/ml* per oocyte; Group B: 150–200 *pg/ml* per oocyte; and Group C: >200 *pg/ml* per oocyte. The clinical data were collected including maternal age, etiology of infertility, type of assisted reproductive treatment (IVF or ICSI), level of estradiol, and the number of COCs retrieved. The outcomes assessed were the number of mature oocytes, maturation rates, fertilization and embryo formation rates, quality of embryos, pregnancy and live birth rates. This study did not include any frozen embryo transfer (ET) cycles. Also, 49 out of 205 cycles were excluded because of the risk of OHSS. However, it should be noted that their data were included until embryo formation, and then excluded because of the risk of OHSS.

### Stimulation protocol:

The patients were stimulated with the standard GnRH antagonist protocols (17). In the antagonist protocol, 150 *IU/day* of follicle-stimulating hormone (FSH Gonal F, Serono, Switzerland) was administered on day two of the menstrual cycle. When at least one follicle reached 13 *mm*, 0.25 *mg* of a GnRH antagonist (Cetrotide, Merck-Serono, Germany) was initiated and continued until the day of human chorionic gonadotropin (hCG) injection. When proper follicular development was viewed on the transvaginal ultrasound, recombinant hCG (Ovitrelle, Merck-Serono, Germany) was administered to trigger final maturation and ovulation. Ultrasound-guided oocyte collection was performed using a single lumen aspiration needle (Wallace; Smiths Medical International, UK) after 36 *hr*. ET was performed 2–3 days after oocyte retrieval via the vaginal route.

### Fertilization and embryo assessments:

The fertilization was assessed 16–18 *hr* after ICSI/IVF, and the embryo cleavage evaluation was done 24 *hr* later. Embryo morphology was evaluated on the basis of the number of blastomere, and the percentage of fragmentation on days 2 and 3 (18). ET was done on days 2 or 3, and the embryos of the patients with risk of OHSS were frozen.

### Statistical analysis:

The statistical analyses were performed using SPSS 20 (SPSS Inc., Chicago, IL, USA). One-way analysis of variance (ANOVA), chi-square and Kruskal-Wallis tests were performed. Logistic regression models were used to determine the association between the ART outcome, and the dependent variables. The appropriate differences were considered significant at p< 0.05.

## Results

A total of 250 ART cycles were evaluated. The mean female age was 29.92±3.9 years, and the indications of ART consisted of male factor (n=96), polycystic ovary syndrome (n=41), ovarian factors (n=23), endometriosis (n=18), tubal factors (n=16), and idiopathic disease (n=11).

There were significant differences between the groups based on the E2 levels with respect to the number of COCs and MII oocytes. Also, the number of good quality embryo was more significant in group C compared to group A and B (Table 1). In group C, the rates of chemical pregnancy (54.1%), clinical pregnancy (50%) and live birth (45.8%) were significantly higher compared to the other groups (Table 1).

**Table 1.** Comparisons of laboratory and clinical outcomes between different groups

**Parameters**	**Group A (n=74)**	**Group B (n=90)**	**Group C (n=41)**	**p**
Number of COCs retrieved ^*^	7 (1–24) 7.27±4.43	11 (2–33) 12.91±6.49	15 (5–44) 18.05±8.2	^a^ <0.0001^#^
				^b^ <0.0001
				^c^ 0.001
Number of MII oocytes ^*^	6 (1–18) 6.16±3.9	9 (1–30) 10.77±5.81	14 (5–39) 15.17±7.43	^a^ <0.0001^#^
				^b^ <0.0001
				^c^ 0.001
**Maturation rate (%)**	84.12±20.9	82.46±16.9	85.05±15.5	0.29^~^
**Fertilization rate (%)**	71.02±23.7	69.57±24.2	65.31±17.6	0.22^~^
**Embryo formation rate (%)**	89.58±18.1	83.84±19.7	89.66±16.3	0.869^#^
**Number of good quality embryos**	2.27±2.27	3.68±3.50	6.73±4.90	<0.0001^#^
**Good quality embryos (%)**	57.38±36.6	57.46±33.9	71.19±30.2	0.1^#^
**Chemical pregnancy rate (%)**	39 (25/64)	30.8 (21/68)	54.1(13/24)	^a^ 0.236^$^
				^b^ 0.033
				^c^ 0.001
**Clinical pregnancy rate (%)**	31.2 (20/64)	20.5 (14/68)	50 (12/24)	^a^ 0.09^$^
				^b^ 0.006
				^c^ <0.0001
**Live birth rate (%)**	12.5 (8/64)	11.7 (8/68)	45.8 (11/24)	^a^ 0.852^$^
				^b^ <0.0001
				^c^ <0.0001

Group A, E2: ≤1500 (*pg/ml*); Group B, E2: 1500–3000 (*pg/ml*); Group C, E2: >3000 (*pg/ml*). COC: Cumulus oocyte complex, MII: Metaphase II.

* Data are presented as median (min- max), and mean ±SD.

a: Difference between group A and B,

b: Difference between group A and C,

c: difference between group B and C.

#: Kruskal-Wallis test.

~ ANOVA test.

$: Chi-square test

The data also showed the numbers of retrieved COCs, MII oocytes, and fertilized oocytes were significantly higher in group C with >200 *pg/ml* E2 per oocyte than group A and B (Table 2). The rate of the good quality embryo was not significant between groups. Moreover, according to E2/oocyte ratio, the rate of live birth was higher in group C compared with group A (18.3%, p=0.04), and group C (29.7%, p<0.0001) (Table 3).

**Table 2.** Laboratory characteristics of groups with different E2/oocyte ratios

**E2/oocyte ratio**				

**Variables**	**Group A ≤150 *pg/ml***	**Group B 150–200 *pg/ml***	**Group C >200 *pg/ml***	**p**
**Number of cycles**	83	70	52	
**Level of E_2_ on day of HCG administration (*pg/ml*)**	1741.18±1003.81	1569.81±828.4	3575.62±2009.4	^a^ 0.692
				^b^ <0.0001
				^c^ <0.0001
Number of COCs retrieved^*^	16 (6–44)	7 (1–22)	9 (2–33)	^a^ <0.0001
				^b^<0.0001
				^c^ 0.049
Number of MII oocytes ^*^	14 (1–39)	6 (1–15)	8 (1–26)	^a^ <0.0001
				^b^ <0.0001
				^c^ 0.008
Number of 2PN^*^	8 (0–27)	4 (0–11)	6 (1–17)	^a^ <0.0001
				^b^ 0.003
				^c^ 0.056

E2: Estradiol, HCG: Human chorionic gonadotropin, MII: Metaphase II, PN: Pronucleus.

* Data are presented as median (min- max).

a: Difference between group A and B,

b: difference between group A and C,

c: difference between group B and C. Kruskal-Wallis test

**Table 3.** Reproductive outcomes in groups with different E2/oocyte ratios

**E2/oocyte ratio**				

**Variables**	**Group A ≤150 *pg/ml***	**Group B 150–200 *pg/ml***	**Group C >200 *pg/ml***	**p**
**Maturation rate (%)**	85.38±15.2	80.1±20.7	85.39±18.5	0.28~
**Fertilization rate (%)**	66.09±23.5	74.52±22.8	67.16±21	0.057~
**Embryo formation rate (%)**	84.36±18.3	86.95±20.4	91.59±15.9	^a^ 0.07^#^
				^b^ 0.002
				^c^0.21
**Good quality embryos (%)**	56.61±31.4	61.89±36.4	63.65±36.7	0.27^#^
**Chemical pregnancy rate (%)**	46.6 (28/60)	23.7 (14/59)	43.2 (16/37)	^a^0.001 ^$^
				^b^ 0.57
				^c^0.004
**Clinical pregnancy rate (%)**	36.6 (22/60)	16.9 (10/59)	40.5 (15/37)	^a^ 0.001 ^$^
				^b^ 0.6
				^c^ <0.0001
**Live birth rate (%)**	18.3 (11/60)	8.4 (5/59)	29.7 (11/37)	^a^ 0.03^$^
				^b^ 0.04
				^c^ <0.0001

A: Difference between group A and B,

b: Difference between group A and C,

c: Difference between group B and C.

#: Kruskal-Wallis test.

~ ANOVA test.

$: Chi-square test

For further evaluation about the effects of the variables on ART outcomes, logistic regression was done. The effects of female age, level of E2 on day of HCG, number of COCs retrieved, number of MII oocytes, number of embryos at the 2PN stage and good quality embryos rate with clinical pregnancy were all surveyed (R^2^=0.147, p=0.07). Only the number of good quality embryo was marginally a positive predictor (p=0.051, odds ratio=1.7,95% CI:0.99–3.05, B=0.5). About the effect of above parameters and live birth, logistic regression (R^2^=0.19, p=0.007) showed number of good quality embryos was a positive predictor for the live birth rate (odds ratio=2.03, 95% CI:1–4.1), but level of E2 on day of HCG was a negative predictor (odds ratio=0.99, 95% CI:0.99–1).

## Discussion

Successful implantation depends on the synchronized development of both embryos and endometrial receptivity (19). So, E2 is the key factor that may affect these parameters and a few publications about this factor are available (5, 13, 16, 20–22). The supraphysiological levels of E2 unavoidably occurs during COH followed by the development of multiple ovarian follicles (1). The effect of E2 levels on the outcomes of IVF-ICSI has remained controversial (19). In this study, the effect of both total serum E2, and E2/oocyte ratio on IVF-ICSI outcomes was assessed.

Our results showed that with elevation of serum E2, an increase in the number of MII oocytes was noticed. In addition, the number of good quality embryo increased significantly. It is logical to have an increase in COC count by elevation of estradiol level. Therefore, with increasing the number of oocyte, probably the number of good quality embryo will increase. However, the rates of embryo formation and good quality embryo were not different between groups. So, the high peak in E2 level was not associated with a lower ratio of high quality embryo formation. These data suggest that the developmental quality of oocytes is not impaired by elevated E2. Furthermore, in contrast to the previous findings (3, 9, 23), best quality embryos, the clinical pregnancy and live birth in the group with the highest peak of E2 (Group C) were obtained.

Similar to our findings, Kara et al. (21) showed that higher pregnancy and implantation rates may be achieved when the serum E2 levels increase. In addition, they did not observe the negative effect of supraphysiological serum E2 level on IVF-ICSI outcome. In our study, chemical and clinical pregnancies, as well as live birth rates, were increased with elevated level of E2. But logistic regression model demonstrated E2 is a negative predictor for final outcome, while, the number of good quality embryo was a positive predictor. Maybe, increasing E2 causes an increase in the number of good quality embryos, but has detrimental effect on endometria, and adversely affects the implantation (24, 25). It is suggested to perform embryo freezing to avoid the detrimental effect of elevated E2 on the endometrium and implantation. Mittal et al. (22) showed that with an increase in serum E2, a higher number of oocytes were retrieved and more mature oocytes were seen with an increase in number of cryopreserved embryos, but no differences in overall pregnancy rates were reported. However, some studies showed higher serum E2 on the day of hCG resulted in improved pregnancy rates in IVF cycles (5, 26). On the contrary, in some studies (3, 9), increased total serum E2 was correlated with poor pregnancy rate.

While the success of IVF-ICSI outcomes is associated with E2 level, some studies suggested that E2/oocyte ratio is more important to the success of COH cycles (8, 14). Our data clearly showed an association between E2/oocyte ratio and live birth rate. It is concluded that the live birth rate was the highest when E2/oocyte ratio was >200 *pg/ml* per oocyte. As yet, there is no accepted optimal amount for E2/oocyte ratio. Loumaye et al.’s research is one of the first studies about the optimal E2/oocyte ratio (14). They suggested that the optimal ratio of 70–140 *pg/ml* per oocyte has the highest pregnancy rates, and declared that this ratio was predictable for IVF outcome in women treated with GnRH agonist protocol. In our study, the clinical pregnancy and live birth rates were significantly higher in group C (E2/oocyte ratio of >200 *pg/ml* per oocyte). Similar to our study, Var et al. (13) showed an increase in the rates of maturation, fertilization, embryo formation and good quality embryos, when the E2/oocyte ratio increased. In the same study, although good quality embryos and implantation rates insignificantly increased with increase in E2/oocyte ratio, the highest pregnancy rate was recorded in 100–200 *pg/ml* per oocyte group. Overall, they concluded that fertilization rate was significantly the highest in the >200 *pg/ml* per oocyte ratio group, and the lowest in the <100 *pg/ml* per oocyte ratio group. Also, the clinical pregnancy rate was significantly lower in the <100 *pg/ml* per oocyte ratio group than others. Vaughan et al. (27) also evaluated the effect of the serum E2 per oocyte ratio on reproductive outcomes. They reported that clinical pregnancy rate was the highest in the patients with E2/oocyte ratio of 250–750 *pmol/l*, and declined as this ratio increased, independent of the patients’ age. Also, Vaughan et al. reported clinical pregnancy rate was the highest in the patients with E2/oocyte ratio of 250–750 *pg/ml* per oocyte (27).

## Conclusion

In conclusion, supraphysiological levels of E2 resulting from ovarian stimulation procedure had no adverse effects on the quality of the embryos during IVF cycles, even caused an increase in the number of good quality embryo that is a positive predictor for live birth. Also, E2/oocyte ratio data can be a useful adjunct in predicting success rates of IVF cycles.
